# Simultaneity judgment using olfactory–visual, visual–gustatory, and olfactory–gustatory combinations

**DOI:** 10.1371/journal.pone.0174958

**Published:** 2017-04-04

**Authors:** Naomi Gotow, Tatsu Kobayakawa

**Affiliations:** Human Informatics Research Institute, National Institute of Advanced Industrial Science and Technology (AIST), Tsukuba, Ibaraki, Japan; The University of Tokyo, JAPAN

## Abstract

Vision is a physical sense, whereas olfaction and gustation are chemical senses. Active sensing might function in vision, olfaction, and gustation, whereas passive sensing might function in vision and olfaction but not gustation. To investigate whether each sensory property affected synchrony perception, participants in this study performed simultaneity judgment (SJ) for three cross-modal combinations using visual (red LED light), olfactory (coumarin), and gustatory (NaCl solution) stimuli. We calculated the half-width at half-height (HWHH) and point of subjective simultaneity (PSS) on the basis of temporal distributions of simultaneous response rates in each combination. Although HWHH did not differ significantly among three cross-modal combinations, HWHH exhibited a higher value in cross-modal combinations involving one or two chemical stimuli than in combinations of two physical stimuli, reported in a previous study. The PSS of the olfactory–visual combination was approximately equal to the point of objective simultaneity (POS), whereas the PSS of visual–gustatory, and olfactory–gustatory combinations receded significantly from the POS. In order to generalize these results as specific to chemical senses in regard to synchrony perception, we need to determine whether the same phenomena will be reproduced when performing SJ for various cross-modal combinations using visual, olfactory, and gustatory stimuli other than red LED light, coumarin, and NaCl solution.

## Introduction

Stability of perception in everyday life is preserved by integration of multimodal information. Perception of synchrony in cross-modal combinations plays an important role in maintaining perceptual stability in a continually changing environment.

When researchers examine synchrony perception in cross-modal combinations, they generally perform simultaneity judgment (SJ) task, in which participants report whether two stimuli are presented simultaneously (e.g., [1–5]), or temporal order judgment (TOJ) task, in which participants report the sensory modality of a stimulus that was perceived more rapidly than another one (e.g., [6–10]). In these tasks, cross-modal combinations are presented by varying stimulus onset asynchrony (SOA). Conventionally, the sensory modalities used in these tasks have been limited to physical senses (visual, audio, and tactile sensations), and no studies of this kid have focused on chemical senses (olfactory, and gustatory sensations).

As methods for exploring the environment surrounding the organism, two concepts have been proposed in engineering research: “active sensing” and “passive sensing” [11,12]. Active sensing is defined as searching the environment in a manner that investigates the properties of an object purposefully. To illustrate this situation, using interactions between sensory modalities and the environment, in active sensing the organism watches, gazes at, or carefully observes the appearance of an object with its eyes; strains to hear or listens to the sound from an object with its ears; sniffs the odor that an object gives off through its nose; and moves its hand (or paw) over the surface of an object in order to determine its texture. By contrast, passive sensing means that the organism unexpectedly notices the environment. For example, even if an organism is not going to investigate the properties of an object purposefully, it can look at the object’s appearance, hear sound from the object, smell the odor that the object gives off, or incidentally touch the surface of an object with a part of its body. Cognitive aspects, such as how the organism directs its attention toward the object, concern interactions between sensations and the environment.

As mentioned above, we consider both active sensing and passive sensing to be involved in vision, audition, olfaction, and tactile sensation. On the other hand, in contrast to the other four senses, gustation may be a sensory modality specialized for active sensing. In order for an organism to perceive taste in everyday life, it needs to consume food. As soon as the organism takes food into its oral cavity, it might involuntarily direct its attention to the food.

In chemical sense research, reliable and precise measurement is assured by rigidly controlling gaseous and liquid stimuli. Evens and colleagues [13] proposed the necessary conditions for measuring olfactory evoked potential with high precision: (1) olfactory stimuli must be inserted into an air flow as a pulse in order to prevent stimulation of the trigeminal nerve system by tactile sensation; (2) the olfactory stimulus should reach 70% of its maximum concentration within 50 milliseconds; and (3) air should be at greater than 50% humidity and approximately body temperature. The difficulty of controlling chemical stimuli can be overcome by development of stimulus presentation apparatus that satisfies all their proposals (for olfactory stimulus, [14,15]; for gustatory stimulus, [16,17]).

As mentioned above, although vision is a physical sense, olfaction and gustation are chemical senses. Furthermore, although both passive sensing and active sensing seem to function in vision and olfaction, gustation is likely to function only by active sensing. In this study, we investigated whether these properties of sensory modalities affected perception of synchrony in cross-modal combinations. We performed SJ for three combinations of cross-modal stimuli (i.e., olfactory–visual, visual–gustatory, and olfactory–gustatory combinations), with within-subject design. We used red LED light, coumarin, and NaCl solution as visual, olfactory, and gustatory stimuli, respectively. We determined the temporal distribution of simultaneous response rates in each cross-modal combination for each participant, and calculated approximations on the assumption that these temporal distributions were Gaussian. Using the coefficients of these approximations, we compared the half-width at half-height (HWHH) [18] among three cross-modal combinations. Because HWHH means the extent of the temporal distribution of simultaneous response rate, we defined its value as the temporal resolution of synchrony perception. Furthermore, we compared the point of subjective simultaneity (PSS) [19,20] with the point of objective simultaneity (POS, i.e., SOA = 0 milliseconds) [21] in each cross-modal combination. PSS is equal to SOA value corresponding to the peak on the temporal distribution of simultaneous response rate.

## Methods

### Participant

This study was conducted in accordance with the revised version of the Declaration of Helsinki. All procedures in this study were approved by the ethical committee for ergonomic experiments of the National Institute of Advanced Industrial Science and Technology, Japan. We explained the experiments to each participant in advance of the study, and informed them of their right to cease participation even after their initial agreement to participate; informed written consent was acquired from all subjects. Ten female volunteers without subjective olfactory and gustatory disorders, aged 20–25 (mean age ± standard deviation (SD) = 22.7 ± 1.7 years old), participated in the experiment.

### Stimuli presentation

#### Visual stimulus

In accordance with the previous studies [22,23], green LED light was used as a fixation point and to provide notice of stimulus presentation. Therefore, we selected red LED light, which is the complementary color of green light, as the source of visual stimulus. The luminous body (diameter of 0.24 cm, 57.5 cd/m^2^) derived through an optical fiber was placed about 150 cm in front of the participant. The duration of the visual stimulus was 400 milliseconds per trial.

#### Olfactory stimulus

The odorant was selected on the basis of the following criteria: (1) no stimulation of the trigeminal nerve on the olfactory mucosa, and (2) no unpleasant feeling during smelling. We presented the smell of cherry tree leaves (68.4mM coumarin [Wako Pure Chemical Industries, Tokyo, Japan] dissolved in propylene glycol) as the olfactory stimulus, using an olfactory stimulator developed by Kobal and colleagues (“Olfactometer OM4”: Burghart Instruments, Wedel, Germany). The olfactory stimulus was inserted into an air flow as a pulse. In order to conduct real-time monitoring of stimulus presentation, a high-speed ultrasonic gas sensor [24,25] was placed at the outlet of the olfactory stimulator. The perceived intensity of the olfactory stimulus became approximately ‘moderate’ (3) on a 6-point magnitude scale (odorless: 0, barely detectable: 1, weak: 2, moderate: 3, strong: 4, very strong: 5) [26]. The duration of stimulus presentation was 400 milliseconds, and the flow rate was 7.5 liters per minute. The temperatures of air and olfactory stimulus were adjusted to be equivalent to the temperature in the nasal cavity, i.e., about 36°C. Before starting the measurement, two experimenters confirmed that the perceived intensity of the olfactory stimulus and the temperatures of air and olfactory stimulus at the outlet of stimulator were appropriate for performing SJ. Additionally, white noise was presented at all times during the measurement in order to prevent participant from detecting the timing of stimulus presentation on the basis of the noise of switching between air and the olfactory stimulus.

#### Gustatory stimulus

Tastant was selected on the basis of the following criteria: (1) hydrophilic, and (2) no unpleasant feeling during tasting. We presented a solution of salt (600 mM sodium chloride dissolved in deionized water) as the gustatory stimulus, using an improved version of the gustatory stimulator developed by Kobayakawa and his colleagues [16,17]. The perceived intensity of the gustatory stimulus became was approximately ‘moderate’ (3) on the 6-point magnitude scale. Duration of stimulus presentation was 500 milliseconds, and flow rate was 120 milliliters per minute. Temperatures of deionized water and gustatory stimulus were adjusted to be equivalent to the temperature in the tongue, i.e., about 36°C. Before starting the measurement, two experimenters confirmed that the perceived intensity of the gustatory stimulus and the temperatures of deionized water and gustatory stimulus at the stimulus presentation unit (a Teflon tube in which a small hole of 0.7 × 0.3 cm was drilled into the side) were appropriate for performing SJ.

### Procedure

The experiment was performed in a small room (295 cm in width × 400 cm in depth × 240 cm in height) shielded from outside noise. The door of the room was closed during measurement; a video camera and intercom were placed inside the room so that the experimenter could monitor and communicate with participants from outside.

Sessions consisting of 93 trials were conducted four times for each cross-modal combination. Each participant took part in the experiment over 5 or 6 days, and performed one or two SJ sessions per day. When two SJ sessions were performed in a day, we arranged to present different combinations of cross-modal stimuli.

In all sessions, we placed the luminous body of green LED light derived from an optical fiber about 150 cm in front of the participant. The luminous body of green LED light was adjacent to a luminous body of red LED, which was used as the visual stimulus. The green light was turned on for 7 seconds per trial as a fixation point and a notice of stimulus presentation. In olfactory–visual and olfactory–gustatory combinations, it was possible that the olfactory stimulus would be presented in expiratory or inspiratory phases, resulting in a difference in perceived intensity; therefore, the participant was instructed to stop respirations while the green light was turned on. Additionally, in order to fix the position of their chin, the participant was also asked to hold in their mouth the Teflon tube for presentation of the gustatory stimulus, even in sessions that did not include a gustatory stimulus.

We regarded the gustatory stimulus in the visual–gustatory and olfactory–gustatory combinations, and the visual stimulus in the olfactory–visual combination, as the standard stimuli, whereas we regarded the other stimulus in each combination as the comparison stimulus. The presentation timing of standard stimulus at each trial was adjusted to be ± 500 milliseconds, centered around 3 seconds after the green light was turned on. The inter-stimulus interval was about 20 seconds. Furthermore, in each combination, we prepared 31 steps from −1900 milliseconds (comparison stimulus first: negative sign) to 1900 milliseconds (standard stimulus first: positive sign) as the SOA between the standard and comparison stimuli. These SOAs were controlled automatically by a personal computer. Each SOA was incorporated randomly in a sequence of presented stimuli three times per session, and we prepared six different sequences of presented stimuli in which the same SOA was not repeated successively. We prevented the same sequence from being used repeatedly between sessions in each cross-modal combination.

The participant was asked whether two stimuli, each belonging to different modalities, were presented simultaneously, and they were informed that they were not required to make a quick judgment. The participant was asked to express ‘1’ with their index finger if perceiving two stimuli synchronously, or ‘2’ with their index and middle fingers if perceiving two stimuli asynchronously. They did not display either numbers with their fingers, if they did not perceive either stimulus or both stimuli while the green light was turned on. We observed and recorded the participant’s display with their fingers at all times using a video camera placed in the small room. Furthermore, during measurement we always conducted real-time monitoring of stimulus presentation.

### Analysis

#### Calculation of stimulus arrival time points

Based on a record of the real-time monitoring mentioned above, we calculated the time point at which the presented stimulus arrived at the receptor (see in detail, [27]).

#### Calculation of simultaneous response rate and approximation

We calculated actual SOA values, using the record of real-time monitoring of stimulus presentation. These values were classified into 27 time windows, and the simultaneous response rates were calculated for every time window in each cross-modal combination. Trials in which the participant did not express a judgment with their fingers, as well as trials in which the actual value of the SOA was ≤ −2,050 milliseconds or > 2,050 milliseconds, were excluded from analysis. Out of 3,720 trials acquired for each combination, we analyzed 3,715 (adoption rate of 99.9%) for the olfactory–visual combination, 3,621 (97.3%) for the visual–gustatory combination, and 3,627 (97.5%) for the olfactory–gustatory combination.

Based on the simultaneity judgment responses acquired from participants, we calculated the inter-participant averages of the simultaneous response rates (i.e., values obtained by dividing the number of trials that each participant judged as "simultaneous" by the total number of trials) for all time windows in each cross-modal combination. Furthermore, according to the previous studies [28,29], we assumed a Gaussian distribution for the temporal distributions of simultaneous response rates, we calculated ‘*a*’, ‘*b*’, and ‘*c*’ in *y* = *a*×exp{−(*t*−*b*)×(*t*−*b*)/(2×*c*×*c*)} by the method of least squares.

#### Comparison using HWHH and PSS

We calculated the temporal distributions of simultaneous response rates for every participant in each cross-modal combination. We assumed a Gaussian distribution for the temporal distributions of simultaneous response rates, and calculated approximations by the least-squares method. PSS and HWHH are represented by “*b*” and “*c*” in the coefficients of approximation mentioned above, respectively.

HWHH [18] is calculated by bisecting the interval between two SOA values corresponding to one half of the peak on that distribution. In order to determine whether HWHH differed among cross-modal combinations, we conducted one-way repeated measures analysis of variance (ANOVA) for HWHH with cross-modal combination as a within-subject factor. Multiple comparisons among combinations using Ryan’s method were performed based on the significance of results obtained with ANOVA.

In order to determine whether PSS was equal to POS, we conducted one sample *t*-tests in each cross-modal combination.

## Results

### Calculation of simultaneous response rate and approximation

**Fig 1:**
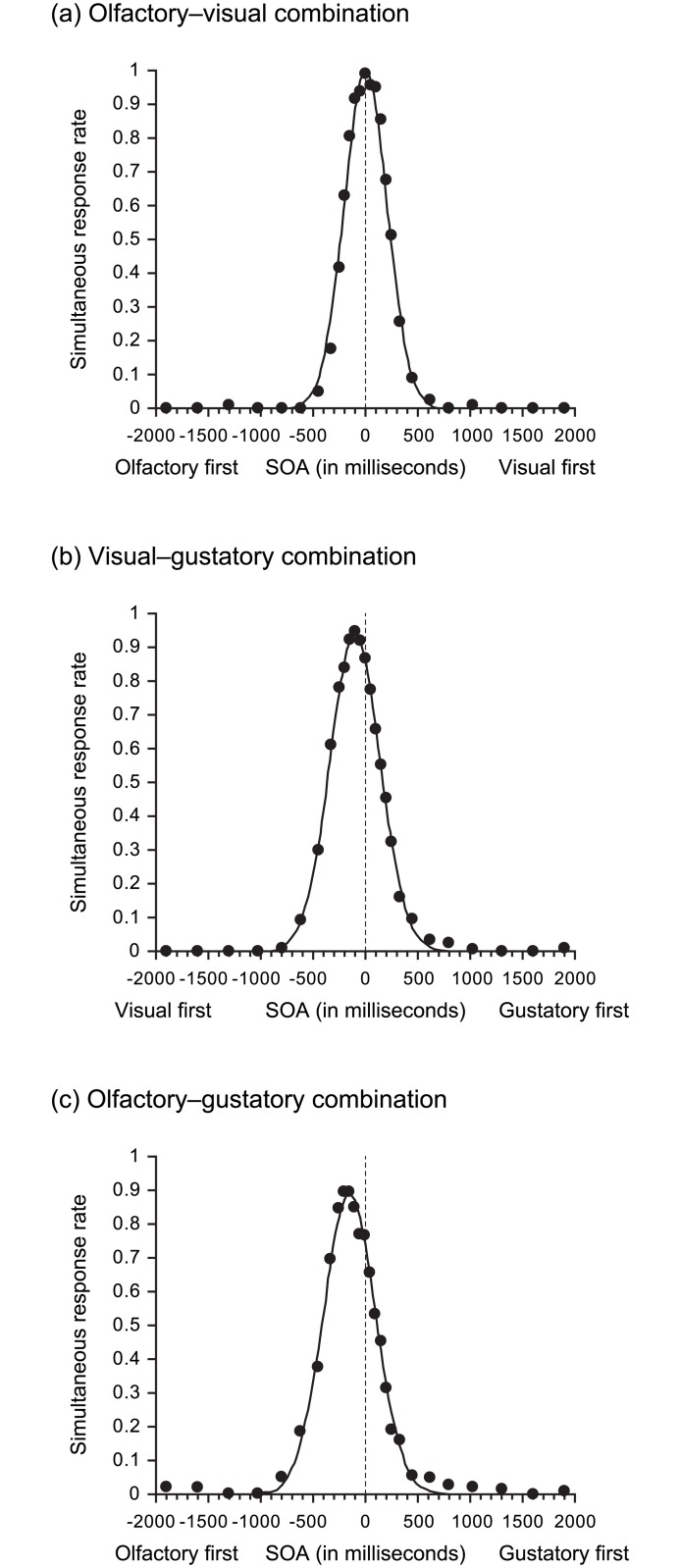
Temporal distributions of simultaneous response rates and approximate curves in each cross-modal combination. We calculated actual stimulus onset asynchrony (SOA) values, using a record of real-time monitoring of stimulus presentation. The actual SOA values were classified into 27 time windows, and the simultaneous response rates were calculated for every time window in each cross-modal combination. Temporal distributions of simultaneous response rates (filled circular dots) and approximate curves (solid line) for olfactory–visual, visual–gustatory, and olfactory–gustatory combinations are shown in (a), (b), and (c), respectively. We assumed that the temporal distributions of simultaneous response rates were Gaussian, and calculated approximations by the least-squares method. The error rates of approximations for olfactory–visual, visual–gustatory, and olfactory–gustatory combinations were 0.4%, 0.1%, and 0.3%, respectively.

Temporal distributions of simultaneous response rates and approximate curves for each cross-modal combination are shown in Fig 1. Approximations were as follows.

y= 1.00×exp{−(t−0.011)×(t−0.011)/(2×0.204×0.204)} [Olfactory–visual combination]

$$y=\;0.94\times \text{exp}\left\{-(t+0.098)\times (t+0.098)/\left(2\times 0.238\times 0.238\right)\right\}\;y=0.94\cdot \text{exp}\left\{-\frac{{\left(t+0.098\right)}^{2}}{2\cdot 0.238^{2}}\right\}\;[\text{Visual–gustatory combination}]$$

y= 0.89×exp{−(t+0.151)×(t+0.151)/(2×0.248×0.248)} [Olfactory–gustatory combination]

In the equations above, the simultaneous response rate and time points (in seconds) are represented by “*y*” and “*t*”, respectively. The error rates of approximations (the value obtained by dividing the sum of squares of the difference between actual simultaneous response rates and theoretical values derived from approximation by the sum of squares of the actual simultaneous response rates) for olfactory–visual, visual–gustatory, and olfactory–gustatory combinations were 0.4%, 0.1%, and 0.3%, respectively. Additionally, Pearson product-moment correlation coefficients between actual simultaneous response rates and theoretical values derived from approximation were *r* > 0.99 in all cross-modal combinations. These results demonstrated the validity of applying the temporal distribution of simultaneous response rates to a Gaussian distribution.

### Comparison using HWHH and PSS

We calculated the temporal distribution of simultaneous response rates for each cross-modal combination for every participant (*n* = 10). We assumed a Gaussian distribution for these temporal distributions, and calculated the approximations by the least-squares method. The error rates of approximations were 0.7–5.5% (mean rate ± SD = 2.61 ± 1.16%) in all sessions. Additionally, Pearson product-moment correlation coefficients between actual simultaneous response rates and theoretical values derived from approximation were 0.95–0.99 (mean rate ± SD = 0.98 ± 0.01) in all sessions.

**Fig 2:**
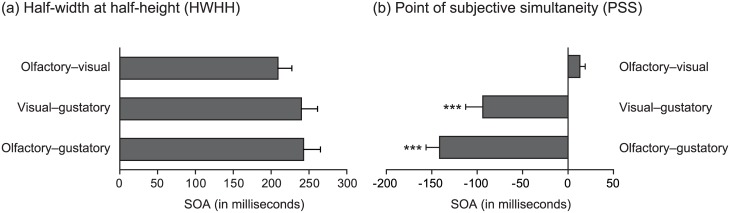
HWHH and PSS of each cross-modal combination. Half-width at half-height (HWHH) of each cross-modal combination are shown in (a). One-way repeated measures analysis of variance (ANOVA) for HWHH with cross-modal combination as a within-factor did not demonstrate significance. The point of subjective simultaneity (PSS) of each cross-modal combination are shown in (b). In olfactory–visual combination, stimulus onset asynchrony (SOA) values with positive sign represented the case that visual stimulus led olfactory stimulus. In visual–gustatory combination, SOA values with negative sign represented the case that visual stimulus led gustatory stimulus. In olfactory–gustatory combination, SOA values with negative sign represented the case that olfactory stimulus led gustatory stimulus. One sample *t*-tests for comparing PSS with the point of objective simultaneity (POS) revealed significant differences for visual–gustatory combination (*t* (9) = 4.83, *p* < 0.001) and olfactory–gustatory combination (*t* (9) = 9.49, *p* < 0.001). Error bars: standard error (*n* = 10). *** *p* < 0.001, * *p* < 0.05.

HWHH in each cross-modal combination were shown in Fig 2(a). One-way repeated measures ANOVA for HWHH did not demonstrate a significance main effect of combination (*F* (2, 18) = 3.01, *p* < 0.1). This result revealed that HWHH did not differ among cross-modal combinations.

PSS in each cross-modal combination were shown in Fig 2(b). One sample *t*-tests for comparing PSS with POS demonstrated significant differences for visual–gustatory combination (*t* (9) = 4.83, *p* < 0.001) and olfactory–gustatory combination (*t* (9) = 9.49, *p* < 0.001). This result revealed that PSS of combination with gustatory stimulus receded significantly from POS.

## Discussion

### Validity of gustatory and olfactory stimulation devices

Needless to say, precise temporal control of stimuli is indispensable for an SJ or TOJ experiments. Physical sensations, i.e., vision, audition, and touch sensation, are relatively easy to control temporally. On the other hand, chemical stimuli such as olfaction or gustation must be handled using special techniques. We already reported the total stimulus system for this chemical SJ experiment [27]; here, we discussed the essential features of olfactory and gustatory stimulators.

We used an “Olfactometer OM4” (Burghart Instruments) for olfactory stimulus, with real–time monitoring using high–speed ultrasonic gas sensor that we developed. Rise time up to 70% of maximum concentration was less than 20 milliseconds through total experiments. This performance satisfied the criteria proposed by Evans and colleagues [13] for measuring chemosensory event–related potentials, and was sufficient for this SJ experiment. Based on the kinetics of odor retention in the nasal cavity, we presented a 400 milliseconds odor stimulus, followed by about 20 seconds rinse. Air flow rate was 7.5 liters per minute, so that the inside of the nasal cavity was washed by > 2 liters fresh air during every trial, which was sufficient to wash out odorant.

We used gustatory stimulator that we developed to measure gustatory event–related magnetic fields and potentials. As described above, the size of the stimulus area was 0.7 × 0.3 cm. Miller [30] reported that the average density of taste buds at the tip of the tongue is 116 per cm^2^, equivalent to ~ 25 taste buds in a 0.7 × 0.3 cm area. In addition, based on the subjective comments obtained from our participants after the end of each SJ session, they succeeded in detecting taste easily. According to the rise time for gustation, the calculated duration for the taste solution’s coverage on this area was about 19 ± 2.5 milliseconds, and this performance also satisfied the criteria proposed by Evans and colleagues [13]. We have already measured both event–related potentials [31] and magnetic fields [16,17] using this taste stimulator. In addition, we showed that the primary gustatory area’s activation linearly increased with the log of NaCl concentration [32], but did not respond to water alone (used as a control condition).

Thus, the stimulator for chemosensation (olfaction and gustation) would be appropriate for SJ measurement.

### Temporal resolution of simultaneous response rate in each cross-modal combination

HWHH did not differ among cross-modal combinations, and the simultaneous response rate reached 0% in an SOA that was approximately 600 milliseconds away from the PSS in all cross-modal combinations. Fujisaki and Nishida [18], who performed SJ using visual, audio, and tactile stimuli, reported that the simultaneous response rate reached 0% in SOAs that were 100–200 milliseconds. Comparison between the results of this study and those of Fujisaki and Nishida [18] revealed that temporal resolution of synchrony perception was lower in cross-modal combinations involving one or two chemical stimuli than in combinations of physical stimuli.

Two causes might explain the results we observed. One possibility is that the HWHH of temporal distribution might have exhibited a higher value in cross-modal combinations involving one or two chemical stimuli than in combinations of physical stimuli because chemical senses (i.e., olfaction and gustation) have lower temporal resolution than physical senses (i.e., audition, vision, and tactile sensation). Fujisaki and Nishida [18] reported that cross-modal combinations of visual and audio stimuli and visual and tactile stimuli exhibited significantly lower temporal resolution than the cross-modal combination of audio and tactile stimuli. They concluded that these results reflected the fact that temporal resolution in the central processing mechanism is lower for visual information than for audio and tactile information. Furthermore, they argued that their results verified the hypothesis that the temporal resolution of synchrony perception in cross-modal combinations depends on the sensory modality with the lower temporal resolution. The second possibility is that information conversion mechanisms in odor and taste receptors might be related. In olfaction, vaporized odorants are separated into molecular. After the resultant molecules are dissolved in viscous liquid of olfactory mucosa, they influence cells bearing olfactory receptors. In gustation, food that is taken into the oral cavity is suspended in by saliva and secretion from von Ebner’s gland, and is ultimately separated into molecules and ions. This molecule or ion affects the surface film (microvillus) of the taste cells, which constitute taste bud. Thus, it takes time to change the electric potential of cell membranes upon presentation of a chemical stimulus. If the time required for this process is not always constant, its variance might reduce the temporal resolution of chemical senses.

### PSS of each cross-modal combination

PSS increased in the following order: olfactory–visual, visual–gustatory, and olfactory–gustatory combinations. The PSS of the olfactory–visual combination was approximately equal to the POS. This finding is consistent with the results of a previous study [18] that performed SJ using visual, auditory, and tactile stimuli. On the other hand, the PSS of visual–gustatory and olfactory–gustatory combinations greatly receded from the POS. Such a result like this has never been observed in simple SJ using combinations of physical stimuli. We inferred that these results might reflect the fact that gustation is specialized for active sensing.

Titchener [33] described the relationship between information processing and attention as fallows: “The object of attention comes to consciousness more quickly than the objects which we are not attending to.” Previous studies on perception of synchrony between sensory information [34–38] reported that stimuli to which participants are directing their attention are processed more rapidly than stimulus to which they are not directing their attention, a phenomenon called the prior entry effect. In some studies of SJ and TOJ using combinations of cross-modal stimuli, participants were asked to direct their attention to one or the other of two stimuli or both stimuli, and PSS was compared among these conditions. On the other hand, Yates and Nicholls [39] suggested that even if the experimental procedure of TOJ is extremely carefully performed, we cannot completely eliminate the possibility of response bias. Here, response bias is defined as the participant’s tendency to report that the sensory modality to which they were directing their attention was perceived faster than another sensory modality to which they were not directing their attention in cases in which they were unable to judge the order of two stimuli in a cross-modal combination. Yates and Nicholls [39] speculated that SJ might reduce the occurrence of response bias more easily than TOJ. Although it is harder for SJ than TOJ to produce prior entry effect, this effect is still observed even in SJ [39,40]. A previous study, in which TOJ was performed using visual and tactile stimuli [41] reported that the PSS under conditions in which the participant directed their attention to only the visual stimulus, to both the visual and tactile stimuli, and to only the tactile stimulus receded from POS in the direction of the visual stimulus first by 22, 53, and 155 milliseconds, respectively. Incidentally, results similar to those of Spence and colleagues [41] were observed in TOJ using audio and visual stimuli [42]. Zampini and colleagues reported that the PSS was shifted when participants directed their attention to a specific sensory modality. Based on the above, we inferred that the prior entry effect might be the result of the participant involuntarily directing their attention more to gustatory stimulus than to the other stimulus in each combination, so that the PSS in SJ with visual–gustatory and olfactory–gustatory combinations shifted in the direction of the gustatory stimulus first In everyday life, consumers must take foods into their oral cavities in order to perceive their tastes. We considered this action to be the trigger that activates active sensing in gustation. When the participant does not know when the gustatory stimulus arrives at their tongue, as in this study, they might need to activate active sensing of gustation by directing their attention involuntarily to their tongue.

In visual–gustatory and olfactory–gustatory combinations, although both PSSs shifted in the direction of visual or olfactory stimuli first, the intervals between POS and PSS differed between these two cross-modal combinations. In other words, we considered that the prior entry effect was produced more strongly in the olfactory–gustatory combination than in the visual–gustatory combination. These results might be explained by the ease of confusing olfactory information with gustatory information in everyday life. For example, olfactory disorder patients who complain of subjective gustatory disorders can be classified into two groups: those perform worse than normal range in a gustatory test, and those who performed normally in a gustatory test. The patients in the latter group are defined clinically as having a “flavor disorder” [43,44]. Kitano and colleagues [44] reported that about half of olfactory disorder patients with subjective gustatory disturbance suffer from a flavor disorder. In everyday life, although even healthy people might confuse input of olfactory information to olfactory mucosa with input of gustatory information to taste cells, as observed in patients with flavor disorder, they do not confuse input of visual information to retina with input of gustatory information to taste cells. We speculated that because participants were likely to confuse olfactory information with gustatory information, they might direct more attention to the gustatory stimulus in the olfactory–gustatory combination than in the visual–gustatory combination when performing SJ. On the base of this speculation, because more attention to gustatory stimulus accelerates processing of gustatory information processing, the olfactory stimulus is forced to be presented faster than visual stimulus. As a result, the interval between the POS and PSS in the olfactory–gustatory combination might become larger than that in the visual–gustatory combination.

### Neural processing mechanism for simultaneity judgment between cross-modal stimuli

The brain regions involved processing of information from each sensory modality have been gradually identified by non-invasive measurements such as functional magnetic resonance imaging, magnetoencephalography, and electroencephalogram. For vision, after visual stimulus received by the retina arrives at the thalamus through the optic nerve, it is projected onto primary visual cortex. It then branches off into the dorsal visual pathway, which responds to motor vision and spatial vision, and the ventral visual pathway, which responds to form vision, and ultimately reaches orbitofrontal cortex [45–49]. For olfaction, after a stimulus received by the olfactory mucosa arrives at the olfactory bulb through the olfactory nerve, it is projected onto piriform cortex, and then branches off to orbitofrontal cortex and thalamus [50–54]. For gustation, after a stimulus received by a taste cell on the taste buds of fungiform papillae, which are distributed on the frontal one-third of the tongue, arrives at the thalamus through the chorda tympani nerve, it is projected onto the primary gustatory area, and ultimately reaches orbitofrontal cortex [16,17,55–57]. Psychophysical and neuroimaging studies using cross-modal stimulus have identified orbitofrontal cortex as the brain region concerned with interaction among sensory modalities [58–61]. Furthermore, recording from single neurons of macaque verified that visual, olfactory, and gustatory information converges in orbitofrontal cortex [62]. Thus, this brain region functions in both unimodal information processing such as visual, olfactory, and gustatory stimuli and in bimodal (or multimodal) information processing such as olfactory–visual, visual–gustatory, and olfactory–gustatory combinations. In the near future, we should investigate whether orbitofrontal cortex is activated when performing SJ.

### Effect of orthonasal and retronasal olfaction on simultaneity judgment

Olfaction is the only dual–sensory modality that perceives both odorants in the external world and those in the body (i.e., the mouth) [63]. Orthonasal olfaction occurs when an odorant molecular is delivered via the nares to olfactory epithelium, whereas retronasal olfaction occurs when an odorant molecular is delivered from the oral cavity via the nasopharynx and posterior choanae to olfactory epithelium in the olfactory cleft [64]. Orthonasal and retronasal olfaction are processed in different brain regions [65,66]. Small and colleagues [51] measured brain activity by functional magnetic resonance imaging during orthonasal and retronasal presentation of four odors (butanol, farnesol, lavender, and chocolate). The results revealed that the brain regions that were activated depended on the routes of odor presentation only when chocolate, a food odor, was used. More specifically, orthonasal presentation increased brain activity in the insula/operculum, thalamus, hippocampus, amygdala, and caudolateral orbitofrontal cortex, whereas retronasal presentation increased activity in the perigenual cingulate and medial orbitofrontal cortex. The differences between orthonasal and retronasal olfaction have been described in previous studies of event-related potential [67–69], detection [70,71], identification [72,73], and perceived intensity [74,75]. In this study, olfactory stimulus was presented orthonasally. Based on the previous studies, we might obtain different results regarding simultaneity judgment for cross-modal combinations involving an olfactory stimulus, depending on whether it is presented orthonasally or retronasally.

## Conclusion

Vision is a physical sense, whereas olfaction and gustation are chemical senses. When we approach the properties of sensory modalities from a different standpoint, we might reasonably suppose that both active and passive sensing function in vision and olfaction, whereas only active sensing functions in gustation. In order to examine the effect of each sensory property on synchrony perception, we had our participants perform SJ using three cross-modal combinations of olfactory–visual, visual–gustatory, and olfactory–gustatory stimuli. We used red LED light, coumarin, and NaCl solution as visual, olfactory, and gustatory stimuli, respectively. We determined the temporal distribution of simultaneous response rates in each cross-modal combination for each participant, and calculated approximations on the assumption that these temporal distributions were Gaussian. Furthermore, using the coefficients of these approximations, we compared HWHH among cross-modal combinations, and compared PSS with POS in each combination. This result revealed that there were no significant differences in HWHH in all paired cross-modal combinations. The HWHH of three cross-modal combinations were higher than the HWHH of cross-modal combinations of physical stimuli obtained in a previous study [18], so we considered that cross-modal combinations involving chemical stimuli might have a low temporal resolution of synchrony perception. The PSS increased in the following order: olfactory–visual, visual–gustatory, and olfactory–gustatory combinations. The PSS of the olfactory–visual combination was appropriately equal to the POS, as in the case of SJ using three physical stimuli (visual, audio, and tactile stimuli). On the other hand, the PSS of visual–gustatory and olfactory–gustatory combinations receded significantly from the POS.

This study is the first report using the method established by Gotow and Kobayakawa [27] to perform SJ for olfactory–visual, visual–gustatory, and olfactory–gustatory combinations. Therefore, in order to generalize these results as the specific to chemical senses (especially gustation) in regard to synchrony perception, we need to verify whether the same phenomena will be observed when SJ is performed for various cross-modal combinations using visual, olfactory, and gustatory stimuli other than red LED light, coumarin, and NaCl solution.

## Supporting information

S1 File The data underlying the findings in this study. Time points of each stimulus onset, actual SOA values, and responses acquired from participants (i.e., “simultaneous” or “successive”) in olfactory–visual, visual–gustatory, and olfactory–gustatory combinations are shown. We calculated the time points of each stimulus onset and the actual SOA values, using the record of real-time monitoring of stimulus presentation.
(XLSX)
